# Growth and development of children aged 1–5 years in low-intensity armed conflict areas in Southern Thailand: a community-based survey

**DOI:** 10.1186/1752-1505-7-8

**Published:** 2013-04-04

**Authors:** Rohani Jeharsae, Rassamee Sangthong, Wit Wichaidit, Virasakdi Chongsuvivatwong

**Affiliations:** 1 Faculty of Nursing (Establishment Project), Prince of Songkla University, Pattani Campus, Pattani, Thailand; 2 Epidemiology Unit, Prince of Songkla University, Hat Yai Campus, Hat Yai, Thailand

**Keywords:** Children, Growth, Development, Conflict, Thailand

## Abstract

**Background:**

A low-intensity armed conflict has been occurring for nearly a decade in southernmost region of Thailand. However, its impact on child health has not yet been investigated. This study aimed to estimate the prevalence of delayed child growth and development in the affected areas and to determine the association between the violence and health among children aged 1–5 years.

**Methods:**

A total of 498 children aged 1–5 years were recruited. Intensity of conflict for each sub-district was calculated as the 6-year average number of incidents per 100,000 population per year and classified into quartiles. Growth indices were weight-for-age, height-for-age, and weight-for-height, while development was measured by the Denver Development Screening Test II (Thai version). Food insecurity, child-rearing practice, health service accessibility, household sanitation, and depression among the caregivers were assessed using screening scales and questionnaires. Contextual information such as average income and numbers of violent events in each sub-district was obtained from external sources.

**Results:**

Growth retardation was highly prevalent in the area as reported by rates of underweight, stunting, and wasting at 19.3%, 27.6% and 7.4%, respectively. The prevalence of developmental delay was also substantially high (37.1%). Multi-level analysis found no evidence of association between insurgency and health outcomes. However, children in areas with higher intensity of violence had a lower risk of delay in personal-social development (OR = 0.4; 95% CI = 0.2 - 0.9; p-value = 0.05).

**Conclusion:**

Unlike war refugees and internally-displaced persons in camp-like settings, the relationship between level of armed conflict and growth and developmental delay among children aged 1–5 years could not be demonstrated in the community setting of this study where food supply had been minimally perturbed.

## Background

Growth and development in the first few years of life is an important health indicator of children. Severe growth retardation has strong associations with mortality and morbidity among children under the age of five years [1], while developmental delay is strongly associated with impaired psycho-social and intellectual development and learning ability [2,3]. Children residing in areas of war or armed conflicts are at high risk of growth failure and developmental delay [4,5], and high rates of stunting (32–63%) and wasting (16–35%) in children have been found among refugees and internally displaced persons in camp-like settings [4,6-8]. Exposure to armed conflict was found to have a linkage to delayed linguistic development among preschoolers and to be associated with psychological disturbance and poor school achievement among school children [5,9,10].

In Thailand, ongoing armed conflicts in the southern-most provinces have affected Thai security officers, health workers, school teachers, and ordinary civilians alike since January 2004, with an injury rate of 1.0 per 100,000 persons per year and mortality rate of 1.2 per 100,000 persons per year [11]. The intensity of the conflicts in the area is relatively low, various infrastructures have remained intact and only individuals who were directly exposed to armed conflict have been displaced [12]. However, the violence notably disturbs the daily lives of local residents and has the potential to result in community discordance and dysfunction. In addition, other surveys have reported a high prevalence of growth retardation and low school achievement among children in the area [13-15].

Due to the ecological fallacy that undermines prevalence studies, it is difficult to conclude that low-intensity armed conflict is responsible for growth failure and developmental delay among children. Furthermore, no study has previously investigated child growth and development in such a setting. Therefore, this study aims to assess the association between growth and developmental outcomes of community-dwelling children aged 1–5 years and exposure to armed conflict.

## Methods

The study was conducted in rural and semi-urban communities in the provinces of Pattani, Yala, Narathiwat, and Songkhla in southern Thailand.

Sample size calculation was based on the hypothesis that the proportion of delayed growth and development of children in an area with low intensity of armed conflict would be 0.40, while the proportion among children in an area with higher intensity of armed conflict would be 0.60. Using a formula for detection of difference between two proportions, a sample size of 107 cases per group was calculated using R software with Epicalc package. Since the study aimed to compare the effect of armed confict divided into 4 quartiles of intensity, a total of 428 subjects (4 groups with 107 subjects each) would be needed. Assuming a 40% non-response rate due to the nature of the study setting, a final sample size of 600 children (n = 600) was obtained.

Due to security concerns, data collection in home setting was deemed to be unsafe, and local community health centres (CHCs) became the study sites. The CHC is a sub-district-level primary healthcare facility normally staffed by 3–4 public health officers and/or nurses. Fifty out of 405 CHCs in the study area were randomly selected. Staff in each of the selected CHCs provided the researchers with a list of children aged 1 to 5 years who had been living within the service area of the CHC for the past 6 months. Twelve children were then randomly selected from the list, with three children for each age group stratum (12–23 months, 24–35 months, 36–47 months, 48–60 months), resulting in the total number of 12 children per CHC. Children aged 6–11 months were excluded from the sampling frame due to the reluctance of their parents to bring them to the CHC. Such reluctance is the normal child-rearing practice in the area: parents prefer not to take young children outside the home unless in case of necessity.

A list of participants selected by the above mentioned process was given to the health care providers and coordinating volunteers to verify eligibility. Exclusion criteria included physical disability, neurological disorder, psychiatric disorders and chronic medical conditions e.g. autism, HIV/AIDS or other chronic diseases. Those with direct experience of injury or death of family members due to the armed conflict were also excluded because of the imminent effect of such events on the study outcomes.

The primary caregivers of eligible children were informed about the purposes of the study and invited to participate in the study. Both the child and the caregiver were asked to visit the health center for data collection at the appointed time and date. Data were collected from February to May 2010, and most of the data were collected in the morning. Fortunately, no visit was postponed.

Anthropometric measurement for weight and height was taken by trained research assistants using standard instruments. The length of children who were too young to stand was measured in supine position on a length board with 0.1 cm precision. The height of children who were able to stand was recorded without shoes using a height-measuring board with 0.1 cm precision. Children who could not stand were measured for their height in supine position instead of the standing position. The weight of the child was measured by a beam balance scale with 0.1 kg precision. Shoes and clothing were removed to be as light as culturally appropriate before weighting.

The 125-items Denver Development Screening Test II (Thai version) [16] was used to assess personal-social function (25 items), fine motor ability (29 items), language development (39 items), and gross motor ability (32 items). The Denver Test was designed to be used in all children up to 6 years of age. Assessment criteria would vary by the age of the child being examined. The outcome of the test was categorized into normal and suspected delay groups based on the child's level of ability to perform a given task. A child would be considered as having suspected delay when the results showed two or more items under "caution" or one or more item under "delayed". Any child who refused to perform a given task would be categorized as being un-testable. A child classified as having suspected delay or being un-testable would be requested to come for a re-evaluation 1–2 weeks later.

The prominent covariates associated with child growth and development suggested by WHO [17] were taken into account during data analysis. These covariates included immediate factors (e.g. dietary intake and history of illness), underlying factors (e.g. food and health accessibility, sanitation, child-rearing practice), psycho-social factors, and basic factors (socio-economic status of the family and community). Missing covariates were not included in the final analysis. Potential confounders were identified based on the results of previous studies found during literature review.

Date of birth was assessed from the birth certificate. Birth weight was assessed from the child’s health records, the CHC's database, and cross-referenced with the mother's medical history. In case of discrepancy between the 3 sources, the weight that was agreed by 2 sources would be used as the final weight. If all 3 sources were discrepant, the weight from child’s health records was used since it would be the most accurate. History of illness within the previous 12 months was assessed from the child’s health records and through face-to-face interview with the primary caregiver. A 24-hour food recall was assessed by a qualified nutritionist to measure food intake. Food insecurity was measured by the Household Food Insecurity Access Scale (HFIAS) [18], while information on child-rearing practice, health service accessibility, and household sanitation was obtained using a structured questionnaire. The Depression Screening Scale of the Department of Mental Health, Thai Ministry of Public Health [19] was applied to measure depressive disorder among primary caregivers.

Data were coded and computerized (by double entry) using EpiData software version 3.0 [20]. The INMUCAL-Nutrient software 4^th^ edition database [21] was used to compute amount of nutrients from each raw food item that the child consumed. While all values of macro nutrients (carbohydrate, protein, fat) could be searched from the database, it was not possible to get all micronutrient values for local food items. Only micronutrients whose data were available in 80% or more of all food items in the INMUCAL database were included in statistical analysis. Adequacy of each nutrient intake was determined based on the 2003 Dietary Reference Intake for Thai People [22]. Data analysis was carried out using R software.

Prevalences of growth failures were computed with descriptive statistics. Based on 2006 WHO growth standards [23], weight in kilogram, height in centimetre, and age in month of the children were converted to z-scores of weight-for-age (WAZ), weight-for-height (WHZ) and height-for-age (HAZ). Stunting, underweight and wasting were defined as having a z-score of more than 2 standard deviations below the reference for HAZ, WAZ and WHZ, respectively.

The average annual income of each sub-district was obtained from the database of the Thai Ministry of the Interior [24]. The numbers of violent events per month in each sub-district over a 6-year period during January 2004-May 2010 were obtained from the database maintained by the Deep South Coordination Center (DSCC), an impartial non-state actor (NSA) which collects information of victims of violence in the deep south of Thailand [25]. Based on the DSCC data, the intensity of violence in each sub-district was calculated as the average number of incidents per 100,000 population per year and was divided into quartiles.

Multi-level modeling (MLM) analysis using backward elimination procedure was employed to determine the association between the intensity of armed conflict and child growth and development. Adjustments were made for other variables in two levels, i.e. the sub-district level and the individual level. Sub-district level variable was the average annual income per household in the child’s sub-district. Individual-level variables included age group of the child, the child’s birthweight, caregiver’s education, annual household income and ethnicity. The variables which had p-value of less than 0.2 in univariate analysis were included in the full model. ANOVA test was performed to fit the final model.

The study was approved by the Ethical Committee of the Faculty of Medicine, Prince of Songkla University Hat Yai Campus.

## Results

Only 580 children were eligible for the study, as some CHCs did not have 12 children who met the inclusion criteria within their area of responsibility. Among the 580 eligible children, 82 could not be contacted during the time of study, thus data were collected from 498 out of the 580 eligible children, giving a response rate of 86%. One child had missing data on weight and height, leaving a total of 497 children available for analysis of growth indices. Non-response was primarily due to the fact that subjects could not be contacted during the time of the study. It was not feasible to invite these subjects to participate in the study during their second visit due to security constraints of the study setting faced by the researchers.

### Characteristics of study communities, families and caregivers of the children

The total population in all study communities was 334,080. Population per village ranged from 1,153 to 17,991 with the mean of 3,436. Annual household income ranged from 1,300–2,000 USD with a mean of 1,242 USD. During January 2004-May 2010, a total of 1,902 armed conflict events occurred in the 50 study communities with an average rate of 93.8 events per 100,000 population per year. The death and injury rates were 37.3 and 131.1 per 100,000 populations per year, respectively.

Almost all subjects lived in a family with approximately 5–6 members, nearly half of whom had an income below the national poverty line (50 USD/person/month). Although two-thirds (65%) of the caregivers reported no food insecurity problem, more than 60% expressed concern about the rising cost of food following the onset of the armed conflict. Most caregivers were middle-aged mothers of Malay ethnicity whose highest attained education was at the primary school level. In addition to taking care of their children, half of the caregivers also worked in a rubber plantation or a rice paddy or were manual laborers. Nearly half of the caregivers (42%) reported a history of depressive mood.

### Child characteristics, child-rearing practices, and health status

Children’s sex, age, child-rearing practices, developmental stimulation, sickness and health problems by level of armed conflict are reported in Table 1. Data on sex and age were equally distributed across community with different levels of armed conflict. Although exclusive breast-feeding up to 6 months was uncommon, a higher rate was found in sub-districts with higher quartiles of the intensity of conflict. However, an opposite trend was observed for immunization coverage. Most children had received developmental stimulation through various interactions. Nearly all children had a history of illness within the previous 12 months. Almost half of the children had a history of parasitic infection, and one-fifth had a history of being admitted to a hospital. There were no significant differences between intensity of armed conflict and sex, age group, prolonged breast feeding, depression of caregiver, developmental stimulation by singing, storytelling and group playing, number of illnesses during the past 12 and 3 months, and history of asthma. The variables associated with conflict level were exclusive breast feeding, completeness of immunization, developmental stimulation by reading, history of parasitic infection and admission to hospital within the past 12 months.

**Table 1:** Demographic characteristics, child rearing and health status of the children (n = 498)

	**Frequency**	**Quartile level of the intensity of violence, n(%)**				P-value^**^
**Characteristic**	**(%)**	**1**^**st**^	**2**^**nd**^	**3**^**rd**^	**4**^**th**^	
		(≤18.0)^*^	(18.1–33.5)*	(33.6–50.0)^*^	(>50.0^*^)	
*Demographic characteristics*						
*Sex*						0.39
Female	240 (48.2)	49 (41.9)	69 (49.3)	71 (55.0)	51 (45.5)	
Male	258 (51.8)	68 (58.1)	71 (50.7)	58 (45.0)	61 (54.5)	
*Age group*						0.99
12–23 months	116 (23.3)	28 (23.9)	35 (25.0)	30 (23.3)	23 (20.5)	
24–35 months	127 (25.5)	28 (23.9)	38 (27.1)	30 (23.3)	31 (27.7)	
36–47 months	129 (25.9)	31 (26.5)	34 (24.3)	36 (27.9)	28 (25.0)	
48–60 months	126 (25.3)	30 (25.6)	33 (23.6)	33 (25.6)	30 (26.8)	
*Child rearing*						
Exclusive BF^‡^ up to 6 months	79 (16.0)	16 (13.7)	17 (12.2)	23 (18.1)	23 (20.9)	0.02
Prolonged BF^‡^ longer than 12 months	93 (19.7)	19 (17.0)	29 (22.5)	21 (16.8)	24 (22.9)	0.88
Had complete immunization	219 (44.8)	66 (57.9)	61 (43.6)	54 (43.5)	38 (34.2)	0.02
Caregiver found to have depression	208 (41.9)	48 (41.0)	53 (37.9)	53 (41.4)	54 (48.2)	0.77
*Developmental stimulation*^†^						
Singing	420 (84.3)	98 (83.8)	119 (85.0)	113 (87.6)	90 (80.4)	0.69
Story telling	353 (70.9)	76 (65.0)	101 (72.1)	100 (77.5)	76 (67.9)	0.29
Reading	444 (89.2)	101 (86.3)	131 (93.6)	120 (93.0)	92 (82.1)	0.01
Group playing	495 (99.4)	116 (99.1)	140 (100.0)	129 (100.0)	110 (98.2)	0.17
*Sick within 12 months*						0.81
< 2 times	102 (20.5)	27 (23.1)	25 (17.9)	26 (20.2)	24 (21.4)	
2–5 times	215 (43.2)	54 (46.2)	66 (47.1)	50 (38.8)	45 (40.2)	
> 5 times	181 (36.3)	36 (30.8)	49 (35.0)	53 (41.1)	43 (38.4)	
*Health problems within past 12 months*^**†**^						
Parasitic infection	212 (43.2)	32 (27.8)	58 (42.3)	67 (51.9)	55 (50.0)	< 0.001
Asthma	130 (26.1)	32 (27.4)	34 (24.3)	26 (20.2)	38 (33.9)	0.45
Admitted in past 12 months	96 (19.3)	26 (22.2)	26 (18.6)	15 (11.7)	29 (25.9)	0.002
>1-day illness in past 3 months	146 (29.4)	37 (31.6)	46 (32.9)	37 (28.9)	26 (23.2)	0.58

^‡^BF: Breast feeding, ^†^Not mutually exclusive, ^*^ Incidents of armed conflict per 100,000 population per year, ^**^P-value for Chi-square test (Rao-Scott adjustment).

### Prevalence of underweight, stunting and wasting

Table 2 presents the age- and sex-specific prevalence rates of growth retardation represented by underweight, stunting and wasting. The prevalence of underweight was highest among children aged 36–47 months, while for wasting it was highest among those aged 12–23 months, particularly in boys. However, there was no significant association between age group or sex and any form of growth retardation.

**Table 2:** Prevalence of stunting, wasting and underweight by age groups and sex (n = 497)

**Age group**	**Underweight (%)**			**Stunting (%)**			**Wasting (%)**		
	**Total**	**Male**	**Female**	**Total**	**Male**	**Female**	**Total**	**Male**	**Female**
12–23 months	15.5	18.6	12.3	31.0	30.5	31.6	10.3	15.3	5.3
23–35 months	19.7	19.7	19.6	26.0	23.9	28.6	4.7	5.6	3.6
36–47 months	22.7	21.5	23.8	32.0	34.9	29.2	7.8	9.5	6.2
48–60 months	19.0	18.8	19.4	21.4	21.9	21.0	7.1	6.2	8.1
Total	19.3	20.2	18.3	27.6	27.6	27.5	7.4	8.9	5.8

### Prevalence of delayed development

Table 3 shows the prevalence of the four sub-domains of delayed development by age group. Developmental delay tended to be higher in children in older age groups. Language and fine motor delays had the highest overall prevalence (21.1%), while development delay for gross motor skills had the lowest (3.6%). Personal-social development delay had the highest prevalence among children aged 23–47 months.

**Table 3:** Prevalence of delayed development by age group (n = 498)

**Age group**	**Total**	**Prevalence of delayed development, n (%)**				
		**Personal-social**	**Language**	**Fine motor**	**Gross motor**	**One or more domain**
12–23 months	116	4 (3.4)	15 (12.9)	2 (1.7)	4 (3.4)	27 (23.3)
23–35 months	127	19 (15.0)	18 (14.2)	11 (8.7)	3 (2.4)	38 (29.9)
36–47 months	129	20 (15.5)	37 (28.7)	9 (7.0)	5 (3.9)	54 (41.9)
48–60 months	126	13 (10.3)	35 (27.8)	38 (30.2)	6 (4.8)	65 (52.4)
Total	498	56 (11.2)	105 (21.1)	60 (12.0)	18 (3.6)	185 (37.1)

### Effects of community-level intensity of armed conflict on child growth and developmental delay

Table 4 shows the final multi-level analysis of factors associated with growth failure. There was no association between intensity of conflict and growth failure. Having a birth weight of less than 2,500 grams significantly increased the risk of underweight, stunting, and wasting by at least 2 times. Children of Thai ethnicity had lower odds of being underweight or stunted. Age group, caregiver's education, and household and average household income of the sub-district had no significant relationship with growth failure.

**Table 4:** Multi-level model showing associations between armed conflict and underweight, stunting, and wasting

**Determinant**	**Underweight**		**Stunting**		**Wasting**	
	**OR**^**† **^**(95% CI**^**§**^**)**	**P-value**	**OR**^**† **^**(95% CI**^**§**^**)**	**P-value**	**OR**^**† **^**(95% CI**^**§**^**)**	**P-value**^**¶**^
**Sub-district level**						
Violence event rate *(1*^*st*^*quartile = ref.)*						
2^nt^ quartile	1.1 (0.5,2.4)	0.80	0.9 (0.4,1.9)	0.83	0.8 (0.3,3.0)	0.45
3^rt^ quartile	0.8 (0.4,1.8)	0.70	0.6 (0.3,1.4)	0.26	1.2 (0.4,4.0)	0.80
4^th^ quartile	0.9 (0.4,2.0)	0.78	0.8 (0.4,1.8)	0.59	1.4 (0.4,4.8)	0.75
Average household income of the sub-district	1.0 (0.9,1.0)	0.92	1.0 (0.9,1.0)	0.57	0.9 (0.9,1.0)	0.72
**Individual level**						
Age group *(12–23 months = ref.)*						
24–35 months	1.8 (0.8,3.9)	0.14	0.8 (0.4,1.5)	0.50	0.4 (0.1,1.3)	0.13
36–47 months	1.7 (0.8,3.6)	0.16	1.1 (0.6,2.0)	0.75	0.3 (0.1,1.1)	0.07
48–59 months	1.6 (0.7,3.5)	0.27	0.5 (0.3,1.0)	0.05	0.7 (0.2,2.3)	0.56
Birth weight *(*≥*2,500 g = ref.)*						
*<*2,500 g	4.4 (2.2,8.8)	<0.001	2.0 (0.9,3.6)	0.05	2.9 (1.1,7.4)	0.03
Caregiver’s education *(≤ Primary School = ref.)*						
>Primary School	0.7 (0.4,1.3)	0.32	0.8 (0.5,1.2)	0.29	0.7 (0.3,1.7)	0.50
Household income *(< Poverty line = ref.)*						
≥ poverty line	0.6 (0.3,1.1)	0.09	0.6 (0.4,1.0)	0.05	1.7 (0.8,3.8)	0.18
Ethnicity *(Malay = ref.)*						
Thai	0.3 (0.2,0.8)	0.02	0.4 (0.2,0.7)	0.04	-	-

^†^OR: Adjusted Odds Ratio, ^§^CI: Confidence interval, ^¶^P-value for Wald test.

Table 5 shows the results of the multi-level analysis of factors associated with developmental delay. There was no association between intensity of conflict and child development, except for the sub-domain of delayed personal-social development in which children living in areas with conflict intensity in the third quartile were at a lower risk for delay. Age group had significant associations with all sub-domains of developmental delay with higher risks found among children in the older age groups. Children of Thai ethnicity had lower odds of having language and gross motor developmental delays. Caregiver’s education, household and average household income of the sub-district, developmental stimulation and birth weight all had no significant association with any sub-domain of development.

**Table 5:** Multi-level model of effect of armed conflict on language, personal-social, fine motor and gross motor development

**Variables**	**Developmental sub-domain**							
	**Language**		**Personal-social**		**Fine motor**		**Gross motor**	
	**OR**^**† **^**(95% CI**^**§**^**)**	**P-value**^**¶**^	**OR**^**† **^**(95% CI**^**§**^**)**	**P-value**^**¶**^	**OR**^**† **^**(95% CI**^**§**^**)**	**P-value**^**¶**^	**OR**^**† **^**(95% CI**^**§**^**)**	**P-value**^**¶**^
**Sub-district level**								
Violence event rate *(1*^*st*^*quartile = ref.)*								
2^nt^ quartile	0.8(0.4,1.7)	0.67	0.5(0.3,1.2)	0.15	0.5(0.2,1.4)	0.10	0.4(0.1,1.2)	0.11
3^rt^ quartile	0.5(0.2,1.0)	0.05	0.3(0.1,0.8)	0.01	0.7(0.3,1.7)	0.43	0.6(0.2,1.7)	0.36
4^th^ quartile	1.8(0.4,1.6)	0.52	0.4(0.2,0.9)	0.05	0.6(0.3,1.5)	0.33	0.6(0.2,1.7)	0.40
Average household income of the sub-district	1.0(0.9,1.0)	0.05	0.9(0.9,1.0)	0.43	0.9(0.9,1.0)	0.42	0.9(0.9,1.0)	0.89
**Individual level**								
Age group *(12–23 months = ref.)*								
24–35 months	1.3(0.6.2.8)	0.46	5.3 (1.7,16.7)	0.004	2.5(0.8,7.3)	0.10	2.4(0.7,7.9)	0.14
36–47 months	4.0(2.0,8.1)	0.002	9.2 (3.0,28.1)	<0.001	4.6(1.7,12.9)	0.003	6.8(2.4,19.6)	<0.001
48–60 months	3.8(1.8,8.0)	0.004	5.2 (1.6,16.6)	0.006	13.9(5.1,37.6)	<0.001	3.9(1.3,12.1)	0.17
Caregiver’s education *(≤Primary School = ref.)*								
> Primary	1.3(0.8,2.1)	0.32	0.7(0.4,1.3)	0.33	0.6(0.4,1.1)	0.09	1.2(0.6,2.3)	0.54
Household income *(<Poverty line = ref.)*								
≥poverty line	0.7(0.4,1.1)	0.13	0.7(0.4,1.2)	0.21			0.5(0.2,1.1)	0.07
Ethnicity *(Malay = ref.)*								
Thai	0.3(0.2,0.7)	0.004	0.7(0.3,1.5)	0.33	0.5(0.2,1.1)	0.07	0.3(0.1,0.8)	0.03
Birth weight *(≥ 2,500 g = ref.)*								
<2,500 g	0.8(0.4,1.7)	0.61	1.0(0.5,2.3)	0.93	1.4(0.6,2.9)	0.44	0.9(0.4,2.5)	0.99
Stimulated by singing *(Yes = ref.)*								
No	2.1(1.1,3.8)	0.02	-		1.7(0.8,3.4)	0.14	-	

^†^OR: Adjusted Odds Ratio, ^§^CI: Confidence interval, ^¶^P-value for Wald test.

We also ran the model without birthweight included. The results were similar to the model with birthweight, and thus are not shown here.

## Discussion

This is one of the first studies to examine the prevalence of growth failure and developmental delay among children aged 1–5 years and the association with intensity of armed conflict in the community setting. Although the prevalences were high, the only significant association between intensity of violence and growth failure or delay was for the sub-domain of personal-social development.

The prevalences of growth retardations among the study samples were 1.5-2.0 times higher than the WHO’s thresholds for public health concern (underweight ≥10%, stunting ≥ 20% and wasting ≥ 5%) [26], the national average (underweight: 6–9%, stunting: 8–12% and wasting: 4–5%) [13] and other peaceful areas in Thailand with a similar socio-economic status [27,28]. This indicates a moderate to high magnitude of child growth retardation. However, these prevalences were lower than those found in war zones of Africa [5] and Asia [4] where the collapse in the local social and economic infrastructures as well as food insecurity were reported. Although this study found no association between low-intensity of armed conflict and child growth, other common risk factors for child growth retardation were reported, including birth weight and ethnicity. This concurred with other studies, which showed that other known factors such as low birth weight [29], ethnicity and parasitic infection also play an important role in delayed child growth [7].

This study found that children in areas with higher intensity of violence due to the conflict were less likely to have personal-social developmental delay. Such a relationship was similarly found in a longitudinal study in a non-conflict condition [30], in which moderate prenatal anxiety, stress, and depressive symptoms were associated with more advanced postnatal motor and mental development in healthy children.

A high prevalence of depression among the caregivers was found in the study. However, this factor was not associated with the intensity of the conflict itself, but rather with the socioeconomic indicators of the family. This could be due to the fact that none of the caregivers directly experienced physical trauma from the armed conflicts, thus the intensity of the conflict may not be directly linked to anxiety or depressive symptoms.

### Strengths and limitations

Unlike most previous studies which were conducted at refugee camps or in a post-conflict setting, our study was conducted in communities experiencing an on-going armed conflict. Despite the atmosphere of insecurity and confusion, the response rate of this study was high (86%). However, it should be noted that this study excluded many growth-essential micronutrients from the analysis due to incomplete coverage in the INMUCAL database (i.e. content data for such micronutrients were available in less than 80% of all food items), including zinc, iodine, selenium, etc. Such exclusion thus compromised the ability to adjust for the confounding effects of the levels of these micronutrients, which are essential for child growth. Assessment of micronutrient intake could have been more complete if the assessment was supplemented by blood testing and the 24-hour food recall was conducted more than once, which was not feasible in the setting of this study. The cross-sectional design of the study and lacking of a measure of exposure to conflict violence at the individual level may have limited the ability to capture association between exposure to armed conflict and outcomes. The fact that those who experienced injury or death of family members due to direct exposure to the armed conflict were excluded in this study undermined the generalizability of the outcomes. Therefore, the findings of this study are applicable only for those who were exposed to the violence within the context of their community and not those who were the direct casualties or victims of the conflict.

## Conclusions

The study did not find a significant effect of intensity of armed conflict in southern Thailand on child growth and development in the local communities. The existing growth failure and developmental delay may be attributed to the pre-existing problems within the local context rather than the conflict itself. The results imply the need for long-term and socio-culturally appropriate intervention to improve child health in the conflict setting. Further investigations on other important outcomes such as health services, vaccination and school absence should be conducted.

## Competing interests

The authors declare that they have no competing interests.

## Authors’ contributions

RJ designed the study, conducted the review, collected the data, performed statistical analysis, and drafted the manuscript. RS and VC supervised all processes of the study and manuscript writing. WW performed additional literature review, drafted the manuscript and made subsequent modifications. All authors read and approved the final manuscript.

## Authors’ information

RJ (RN, PhD in Epidemiology) is a lecturer at Prince of Songkla University, Pattani Campus.

RS (MD, PhD in Epidemiology) is a senior lecturer at Epidemiology Unit, Prince of Songkla University, Hat Yai Campus.

WW (MSc in Epidemiology) is a researcher at Epidemiology Unit, Prince of Songkla University, Hat Yai Campus.

VC (MD, PhD in Epidemiology) is a professor in Epidemiology Unit, Prince of Songkla University, Hat Yai Campus.
